# A simple, subjective, knee self-evaluation using a single question can be used for a quick assessment of patients undergoing knee surgery

**DOI:** 10.1007/s00402-024-05720-9

**Published:** 2024-12-24

**Authors:** Antonio Klasan, Cedric Donati, Riccardo Compagnoni, Alberto Grassi, Volker Musahl, Jacques Menetrey

**Affiliations:** 1 AUVA UKH Steiermark, Graz, Austria; 2 Johannes Kepler University of Linz, Linz, Austria; 3 Istituto Ortopedico Gaetano Pini, Milan, Italy; 4 Istituto Ortopedico Rizzoli, Bologna, Italy; 5 University of Pittsburgh Medical Center, Pittsburgh, USA; 6 University Hospital of Geneva, Geneva, Switzerland

**Keywords:** Knee, International knee documentation committee, Questionnaire, Outcomes, PROMs

## Abstract

**Purpose:**

The use of patient-reported outcome measures (PROMS) is the cornerstone of clinical research for surgical disciplines, but the use in daily routine can be challenging. One of the most widespread PROMS in knee surgery is the International Knee Documentation Committee (IKDC) questionnaire. The purpose of the present study was to investigate the potential correlation of the IKDC score with a patient’s subjective assessment of the knee using a single question. We hypothesized a correlation between the IKDC score and single question score.

**Methods:**

A prospective, single center study in a comprehensive knee outpatient clinic was performed. Patients willing to participate, presenting in the clinic for the first time were asked to complete the IKDC questionnaire and to answer the question: ”How does your knee know compare to when you were 16 years old, in percentage?” Pearson correlation coefficient and linear regression were used to analyze the IKDC score and the single-question percentage.

**Results:**

After application of inclusion and exclusion criteria, 310 patients were included. The mean age of the participants was 43.3 ± 9.6 years, and 45.2% of the patients were female. Mean IKDC score was 41.11 ± 13.13, compared to the mean score of the single-question 40.90 ± 22.7 (*p* = 0.887). The correlation between the IKDC score and the single-question was significant (*p* < 0.001), however, the person coefficient was 0.460, indicating moderate correlation. The linear regression analysis was also statistically significant (*p* < 0.001), but with a model fitness of r^2^ = 0.211 and B = 0.266. From the 310 patients, 305 (98.4%) found the single-question of more relevance than the IKDC score.

**Conclusion:**

IKDC score and a simple, subjective, knee self-evaluation using a single question demonstrate moderate correlation. The single question can be used for better understanding of discrepancy between the objective score and the patients’ subjective perception of knee function or as a fast, single question proxy score.

## Introduction

Measurement of success of surgical interventions heavily relies on patient-reported outcome measures (PROMS) [1]. The principle idea of PROMS is to provide an objective measurement of patient’s subjective perception of certain aspects directly related to the surgical intervention [2]. Clinical studies are unimaginable without the use of PROMS.

However, PROMS have been criticized for multiple reasons, including inadequate validation [3], difficulty of use in clinical setting [1], and question-burden for patients and participants [4]. They have also been shown to be dependent on patients’ socioeconomic situation and habits [5]. However, in the current lack of simpler and, perhaps, more objective evaluation tools, PROMS remain the most widely used and most widely accepted tool.

One of the most commonly used PROMS in the area of knee surgery is the International Knee Documentation Committee (IKDC) questionnaire [6]. Developed in 2001, it has been used for all aspects of knee surgery including ligament reconstruction [7], meniscus surgery [8], osteotomy [9], fracture fixation [10] and arthroplasty [11]. Consisting of a total of 19 questions, (score/100%) it aims to cover all aspects affected by the surgery [6] and can be regarded as one of the standards [12–14].

Even IKDC, however, has been criticized for being lengthy and poorly representing the patient’s quality of life [15, 16]. The widespread use of IKDC, and the goal of trying to simplify and increase efficiency of clinics, were the reasons why it was used for the present study. The authors believe that one question might simplify the clinical practice and could, potentially, even be used in a research setting.

The purpose of the present study was to investigate the potential correlation of the IKDC score with a patient’s subjective assessment of the knee using a single question: How does your knee know compare to when you were 16 years old, in percentage? We hypothesized a correlation between the IKDC score and single question score.

## Patients and methods

This is an ethically approved (AUVA 13/2023), prospective study performed in a single, tertiary referral center. Around 7000 surgical procedures are annually performed in the center and around 55,000 patients are treated in the center annually. The study was performed in the first author’s specialist comprehensive knee clinic, where elective and semi-acute patients are evaluated. All patients provided consent to participate in the study. The clinic covers all aspects of knee surgery: ligament reconstruction including multiligament knee injuries, meniscal pathology, traumatology and sports traumatology, patella instability, primary and revision arthroplasty.

Patients presenting in the clinic for the first time were screened for participation and after signing the consent, were asked to fill the study questionnaire. The patients were initially blinded to the purpose of the study and the questionnaire.

The patients were asked to fill out the study questionnaire in the waiting room, prior to the consultation. The study questionnaire consisted of the IKDC Score [17] and, finally, the single question: How does your knee now compare to when you were 16 years old, in percentage? An asterisk was added: *Assuming your knee was 100% when you were 16 old.

Any potential questions by the patients were addressed during the consultation, after which the participation was concluded and the study purpose was unblinded to the patient. The patients were then asked to provide feedback if they found the IKDC score or the single-question to be more relevant by answering the question (single choice): “Is the IKDC-questionnaire or the single question more relevant for your condition?”

Age and gender were collected additionally for each patient.

All first-time presenting patients were screened for inclusion. Excluded were patients declining to participate as well as patients who did not completely fill out the questionnaire, including answering the final question.

### Statistical analysis

An a priori power analysis was performed. Using a mean IKDC score of 80%, as an equivalence study, with an equivalence limit of 10%, an alpha of 0.05 and a beta of 0.8, 310 patients were needed to demonstrate equivalence. The difference between mean values was calculated using one-way analysis of variance. The equivalence was evaluated using Pearson correlation coefficient and linear regression analyses, using the following correlation coefficient classification: 0.00-0.10 negligible; 0.10–0.39 – weak; 0.40–0.69 - moderate; 0.70–0.89 – strong; 0.90-1.00 – very strong [18]. Predictive value of the single-question for IKDC Score was calculated using linear regression analysis, with the model fitness, r^2^, and the effect size reported [19]. SPSS 29.0.1.0 (IBM, Armonk, NY, U.S.) was used for the statistical analysis. Statistical significance was set at *p* < 0.05.

## Results

**Fig. 1:**
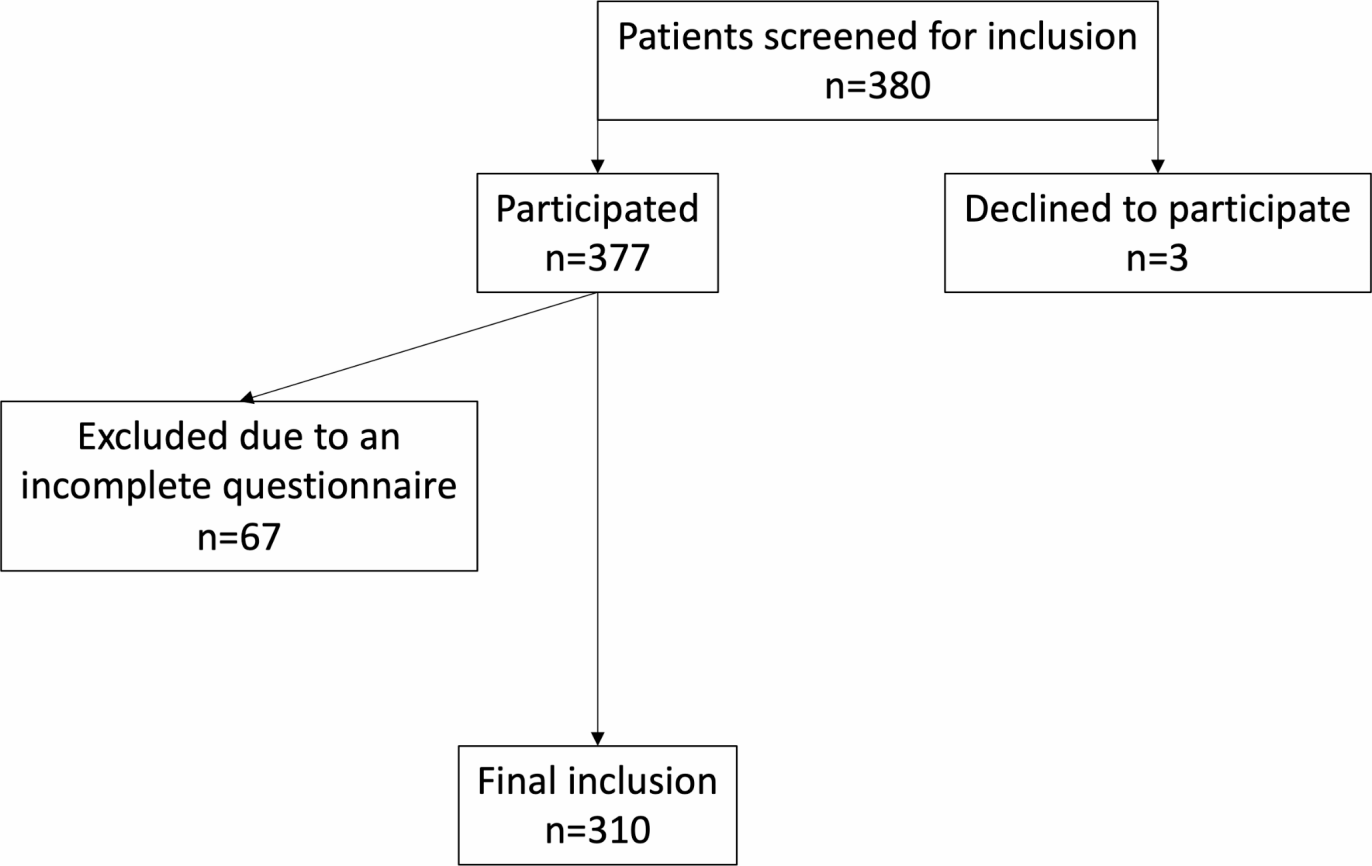
Flow chart of patient inclusion criteria

After application of inclusion and exclusion criteria, 310 patients were included in the study, Fig. 1. The mean age of the participants was 43.3 ± 9.6 years, and 45.2% of the patients were female.

**Fig. 2:**
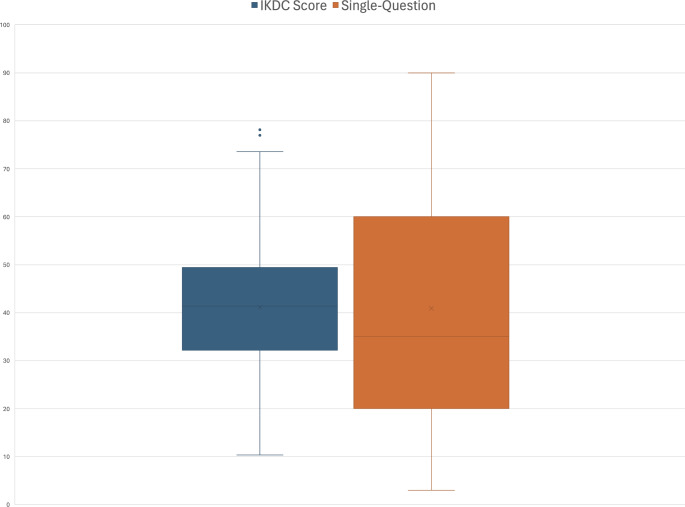
Box and Whisker Plot of the IKDC score and the single question

Mean IKDC score was 41.11 ± 13.13, compared to the mean score of the single-question 40.90 ± 22.7 (*p* = 0.887), Fig. 2.

The correlation between the IKDC score and the single-question was significant (*p* < 0.001), however, the person coefficient was 0.460, indicating moderate correlation.

The linear regression analysis was also statistically significant (*p* < 0.001), but with a model fitness of r^2^ = 0.211 and B = 0.266.

From the 310 patients, 305 (98.4%) found the single-question of more relevance than the IKDC score.

## Discussion

The most important of the present study was a statistically significant, but clinically not-applicable correlation between the IKDC score and the answer to a single-question-questionnaire: “How does your knee know compare to when you were 16 years old, in percentage?”. Also, the predictive value of the single-question for the IKDC score had a very low model of fitness coefficient. The single question cannot, therefore, be interchangeably used with the IKDC questionnaire, but, due to the highly positive feedback, can be used instead to get a better understanding of the patient’s perception of the clinical issue.

The use of PROMS is essential in clinical studies, but has also been shown to improve patient care in general [1]. In the digital age, the surveys have shifted from paper to digital tools such as apps [20]. The advancement of artificial intelligence is facilitating better understanding of PROMS and their components [21]. But, PROMS are not without their challenges, especially in daily care [22]. They shift the consultation towards the PROM and inhibit interaction between the clinical and the patient, the problems can be underestimated, the clinically relevant information is limited [1]. PROMS have also been shown to be heavily reliant on socioeconomic factors, where the weight of the questions does not represent the patients perceived importance of each of the questions [5].

An adequate PROM does not exist, merely a best available one [2]. For measuring success of knee surgery, IKDC is an instrument with good internal consistency, test-retest reliability, content and structural validity, responsiveness and interpretability (no floor and ceiling effects) [23]. It has been widely used for a variety of surgical interventions including meniscal surgery [24], ACL-reconstruction [7] and knee arthroplasty [25]. The reported minimally important clinical difference is around 10.9 points [8]. It has been translated into multiple languages (REFs), which confirms the widespread use. It is not without criticism, though, like any PROM. The content validity and the initial study group have been considered as inadequate [3]. It has been chosen as a comparator in the present study, however, due to its widespread use, with the expectation of close correlation to the single question.

The correlation and the predictive value have been both shown to be statistically significant, but, on the effect-size analysis, have failed. The subjective perception of the knee has shown a significantly wider spread than the IKDC score. Since the role of PROMs is to try to objectively measure specific subjective responses to specifically designed questions [2], it remains unclear which questions should be asked to which patient [2]. In an analysis of the Oxford Knee Score with 12 questions, it has been demonstrated that the patient place different weight on different questions, depending on their socioeconomic status and habits [5]. For instance, one of the questions is kneeling, which has the same weight in terms of points as the question about night pain [5].

To date, no questionnaire takes any of the patient-related variables into account. This study implies that some form of simple, subjective self-evaluation should be added. A discrepancy between simple, subjective evaluation and a complete score might facilitate better understanding of patient expectations [26] and perception of pain [27]. A significant correlation, albeit with only moderate correlation coefficient, might even suggest that a single question is sufficient for an efficient score that might serve as a proxy. The feedback provided by the patients confirms the validity of the single-question.

The Patient Acceptable Symptom State (PASS) has been described as a state where the patient “feels well” after treatment [28]. For patients after an ACL reconstruction, more specifically, 1–5 years after, the PASS threshold for the IKDC score is 75.9 [28]. As the present study only included first visits, prior to any intervention and the PASS score thresholds were calculated postoperatively, it would be an interesting assessment to compare the same single question score postoperatively to the PASS-IKDC threshold.

## Limitations

This is a single center study, the variability in socioeconomic factors and pain thresholds around the world has been well described and might influence the results. Data on surgical interventions and their outcomes has not been collected, however, this was not the purpose of the study. Equally, studies initially validating questionnaires do not measure differences between pre- and postoperative outcomes.

## Conclusion

IKDC score and a simple, subjective, knee self-evaluation using a single question demonstrate moderate correlation. The single question can be used for better understanding of discrepancy between the objective score and the patients’ subjective perception of knee function or as a fast, single question proxy score.

## Data Availability

No datasets were generated or analysed during the current study.

## Abbreviations

PROMS: Patient-reported outcome measures
IKDC: International knee documentation committee
PASS: Patient acceptable symptom state
ACL: Anterior cruciate ligament
